# 4EBP1/2 are active under standard cell culture conditions to regulate the translation of specific mRNAs

**DOI:** 10.1038/s41419-020-03182-6

**Published:** 2020-11-11

**Authors:** Khawla Alasad, Kai Voeltzke, Liron Levin, Guido Reifenberger, Gabriel Leprivier, Barak Rotblat

**Affiliations:** 1 Department of Life Sciences, Ben-Gurion University of the Negev, Beer Sheva, Israel; 2 Institute of Neuropathology, Medical Faculty, University Hospital Düsseldorf, Düsseldorf, Germany; 3 The National Institute for Biotechnology in the Negev, Beer Sheva, Israel; 4 German Cancer Consortium (DKTK), partner site Essen/Düsseldorf, Düsseldorf, Germany

**Keywords:** Proteins, RNA

The *mammalian target of rapamycin* (mTOR) kinase is a nutrient sensor coordinating cellular anabolic and catabolic processes^1^. During favourable metabolic conditions, mTOR promotes protein synthesis by phosphorylating its substrates, including *eIF4E binding proteins 1-3* (4EBP1-3). Upon conditions where mTOR is inactive, the hypo-phosphorylated and active 4EBPs bind to *eukaryotic initiation factor 4E* (eIF4E), competing with the recruitment of eIF4G thus disrupting the formation of the eIF4F complex, in turn leading to inhibition of cap-dependent translation initiation^2^.

It is not known whether 4EBPs regulate mRNA translation in optimal growth conditions, in which mTOR is active and 4EBPs thus phosphorylated and presumed to be inactive. This question is particularly relevant in pathological and physiological conditions where the expression of 4EBPs are up- or down-regulated while mTOR is active.

To assess the activity of 4EBP1/2 under basal cell culture conditions, we used lysates of 4EBP1/2 knockdown (KD) and control scramble shRNA (shSCR) HEK293 cells^3^ to pull down eIF4E and its interacting proteins using m^7^GTP-agarose beads (Fig. 1a). We found more eIF4G bound to eIF4E in KD lysates as compared to shSCR cell lysates (Fig. 1a). This finding was confirmed using 4EBP1/2 WT and double KO (DKO) p53^−/−^ MEFs^3^ (Fig. 1a), suggesting that an active cellular fraction of 4EBPs is detectable in optimal cell culture conditions, even in the presence of active mTOR.

**Fig. 1:**
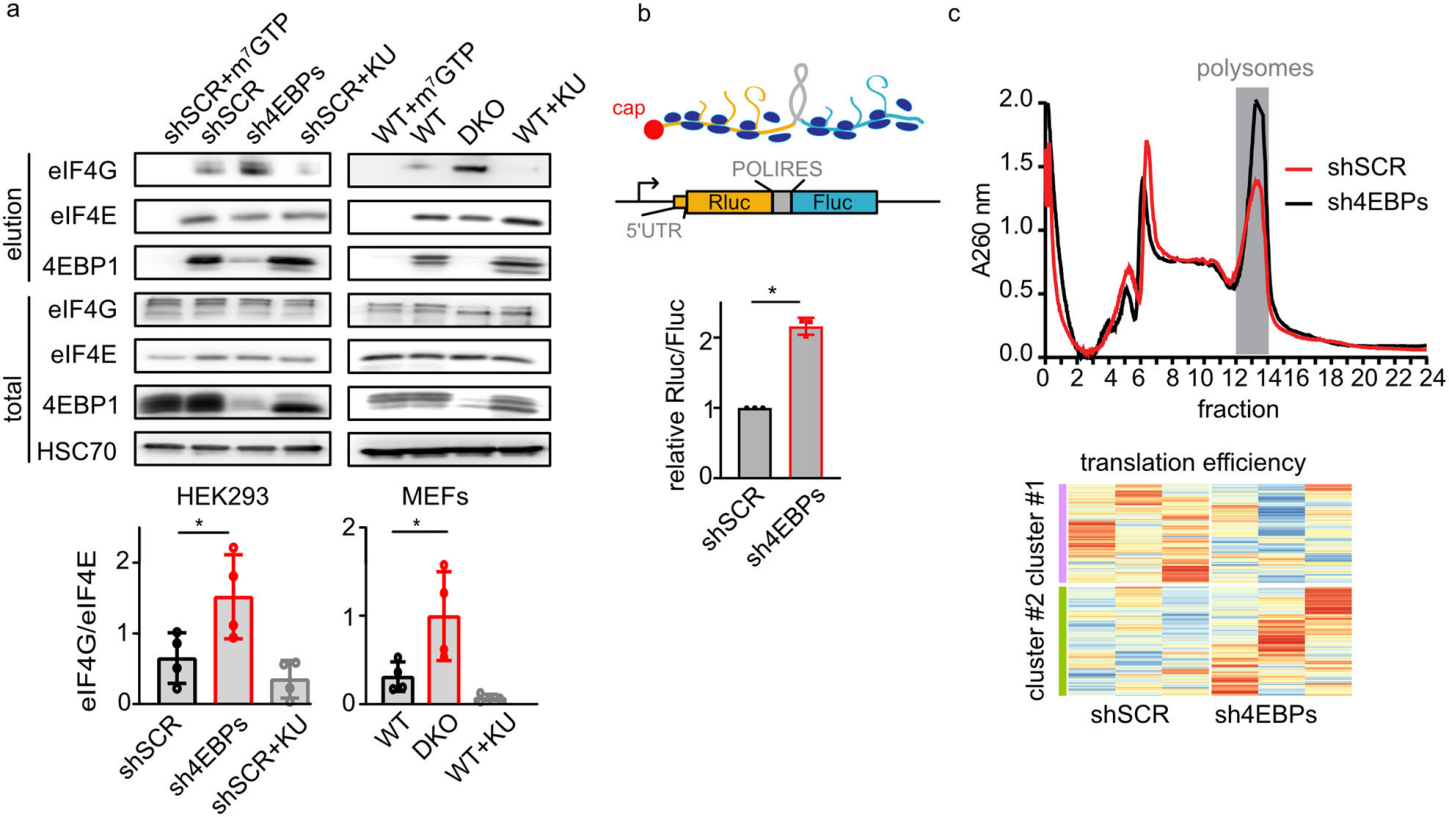
Detection of an active 4EBP1/2 pool under basal conditions. **a** The indicated cells were grown in normal cell culture media, treated or not with 1 μM of the mTOR inhibitor KU-0063794 (KU) for 4h, were lysed and incubated with m^7^GTP coated beads. When indicated, cell lysates were pre-incubated with free m^7^GTP (shSCR+m^7^GTP) to detect nonspecific binding. Eluted and total proteins were analyzed by immunoblot using the indicated antibodies. Protein levels of m^7^GTP bound eIF4G and eIF4E were quantified using ImageJ and presented in a bar graph; *p < 0.05. **b** Scheme of the bicistronic Luciferase reporter; Rluc is driven by cap-dependent mRNA translation through an artificial 5′UTR, Fluc is produced by cap-independent mRNA translation through a poliovirus IRES (POLIRES). HEK293 shSCR and sh4EBPs transfected with pcDNA3-RLUC-POLIRES-FLUC bicistronic vector were grown in normal media. 24h post-transfection, cells were lysed and levels of Rluc and Fluc were sequentially measured. Results are expressed as Rluc/FLuc ratio (*n* = 3 biologically independent experiments). Data are reported as means ± SD with indicated significance (*p < 0.05). **c** HEK293 shSCR and sh4EBPs cells were lysed, separated on non-linear sucrose gradients and polysome profiles were generated by measuring absorbance at 260 nm. Polysomal fractions, highlighted in grey, were collected together with total mRNA. mRNA was then extracted and sequenced. Heat map representation of transcripts whose TE is significantly different between the HEK293 shSCR and sh4EBPs cells, highlighting the three biological replicates.

However, we did not find a statistically significant effect of the active 4EBPs fraction on overall protein synthesis using AHA labelling^4^ under basal conditions, although there was a trend towards increased protein synthesis in DKO cells (Fig. S1a). Nevertheless, using a bicistronic reporter vector in which *Renilla* luciferase (Rluc) is translated in a cap-dependent manner, while Firefly luciferase (Fluc) is translated in a cap-independent manner (Fig. 1b)^5^, we found that KD cells exhibited a significantly higher Rluc/Fluc ratio as compared to controls (Fig. 1b). These data suggest that while 4EBP1/2 restrict cap-dependent translation in normal cell culture conditions, this has minimal impact on overall protein synthesis, pointing to a selective regulation of mRNA translation.

We then identified transcripts whose translation is selectively influenced by 4EBP1/2 under basal conditions, by performing polysome profiling using a non-linear sucrose gradient (the Larsson protocol)^6^ (Fig. 1c). Total and polysomal mRNA, obtained from KD and shSCR cells, were identified and quantified by RNAseq. Analysis of total mRNA expression showed that only 26 genes were differentially expressed between KD and shSCR cells (Fig. S1b and Supplementary Table 1). We calculated the translation efficiency (TE) of each mRNA as the ratio between polysomal and total mRNA levels in KD and shSCR samples (Fig. S1c) and found 516 transcripts with lower TE in KD cells (cluster #1) and 569 transcripts whose translation was increased in KD cells (cluster #2) (Supplementary Table 1). KEGG analysis of transcripts whose TE was affected by 4EBP1/2 identified pathways previously linked to 4EBP1/2 functions including ribosomes, oxidative phosphorylation, metabolic pathways and neurodegeneration (Fig. S1d)^3,7,8^. Overall, these data show that 4EBP1/2 selectively affect the translatome in normal cell culture conditions.

## Supplementary information

Supplementary information

Supplementary figure

Supplementary Table 1

## References

[1] Kim J, Guan K-L (2019). mTOR as a central hub of nutrient signalling and cell growth. Nat. Cell Biol..

[2] Haghighat A, Mader S, Pause A, Sonenberg N (1995). Repression of cap-dependent translation by 4E-binding protein 1: competition with p220 for binding to eukaryotic initiation factor-4E. EMBO J..

[3] Dowling RRJO (2010). mTORC1-mediated cell proliferation, but not cell growth, controlled by the 4E-BPs. Science.

[4] Marciano, R., Leprivier, G. & Rotblat, B. Puromycin labeling does not allow protein synthesis to be measured in energy-starved cells correspondence. *Cell Death Dis.*10.1038/s41419-017-0056-x (2018). 10.1038/s41419-017-0056-x PMC5833866 29348556

[5] Tsukumo Y, Sonenberg N, Alain T (2016). Transcriptional induction of 4E-BP3 prolongs translation repression. Cell Cycle.

[6] Liang S (2017). Polysome-profiling in small tissue samples. Nucleic Acids Res.

[7] Thoreen CC (2012). A unifying model for mTORC1-mediated regulation of mRNA translation. Nature.

[8] Hsieh AC (2012). The translational landscape of mTOR signalling steers cancer initiation and metastasis. Nature.

