# Supplementary material for: 4EBP1/2 are active under standard cell culture conditions to regulate the translation of specific mRNAs
Source: Cell Death Dis. 2020 Nov 11;11(11):968. doi: 10.1038/s41419-020-03182-6 (PMC7659004; doi:10.1038/s41419-020-03182-6)
Supplement: Supplementary file 1 — Supplementary information [file 41419_2020_3182_MOESM1_ESM.docx]

# Supplementary material

# 4EBP1/2 are active under optimal cell culture conditions to regulate the translation of specific mRNAs

Khawla Alasad^1^, Kai Voeltzke^2^, Liron Levin^3^, Guido Reifenberger^2,4^, Gabriel Leprivier^2^, Barak Rotblat^1,3†^

# Figures

SUP Figure 1.

a. The indicated cells grown in normal cell culture media were left untreated (-AHA) or pulsed with 50 μM AHA (+AHA) for 4 hours to allow its incorporation into newly synthetized proteins. Cell lysates were subjected to a Click reaction in the presence of biotin-alkane to label AHA containing proteins. The amount of biotin labeled proteins and the indicated proteins were determined by immunoblotting using streptavidin-HRP and the indicated antibodies. Protein levels were quantified using ImageJ and presented in a bar graph; not significant (ns) p>0.05.

b. Total mRNA from HEK293 shSCR and sh4EBPs cells were sequenced and analyzed by RNAseq (n = 3 biological replicates). Volcano plot shows that the expression levels of very few mRNA were significantly affected by 4EBPs depletion.

c. TE analysis of transcripts obtained from HEK293 shSCR versus KD cells identified two clusters.

d. KEGG analysis of transcripts belonging to cluster 1 and 2 highlights biological pathways over represented in the group of transcripts whose translation are regulated by 4EBPs.

# Tables

SUP Table 1. RNAseq results; TE results

# Methods

## Cell culture

Human embryonic kidney 293 (HEK293) control (shSCR) and stable knock down for 4EBP1/4EBP2 (sh4EBPs) cell lines, WT (p53^-/-^) and 4EBP1/4EBP2 DKO (p53^-/-^) mouse embryonic fibroblasts (MEFs) were kind gifts from Prof. Nahum Sonenberg (McGill University, Canada)^1^. All the cell lines were cultured in Dulbecco's Modified Eagle Medium (DMEM) (01-056-1A, Biological Industries, Kibbutz Beit-Haemek, Israel) containing 10% fetal bovine serum (FBS) (04-007-1A, Biological Industries), 1 mM sodium pyruvate solution (03-042-1, Biological Industries) and antibiotic–antimycotic 100X (bioWORLD, Ohio), at 37 °C and 5% CO_2_. For each experiment, media was replaced with fresh full media one hour prior harvesting cells.

## Compounds

KU-0063794 (sc361219) was purchased from Santa Cruz Biotechnology, Inc, Texas and cycloheximide (R750107) from Sigma-Aldrich, Missouri.

## Cap-dependent / cap-independent translation assay

pcDNA3-RLUC-POLIRES-FLUC bicistronic vector (gift from Prof. Nahum Sonenberg [Addgene plasmid # 45642])^2^ was transiently transfected into HEK293 using Calfectin (SignaGen, Rockville, MD). 250 ng of bicistronic vector was transfected per well of a 12-well plate (Costar). Media was replaced one hour prior harvesting. 24 hr after transfection, cells were lysed and analyzed for *Renilla* Luciferase (Rluc) and Firefly Luciferase (Fluc) activity using the Dual Luciferase Reporter Assay System (Promega) following manufacturer’s instructions. Luciferase activity was expressed as the ratio of Rluc/Fluc to indicate the relative level of cap-dependent to cap-independent translation and to normalize Rluc activity between transfected cell lines.

## Overall protein synthesis rate assay

To quantify levels of newly synthesized proteins, 50 μM of azidohomoalanine (AHA) (C10102, Thermo Fisher Scientific, Massachusetts) was added to the medium and incubated with the cells for 4 hours. Cells were then washed with ice-cold PBS, collected and lysed with EDTA-free RIPA lysis buffer (150 mM NaCl, 50 mM Tris [pH 8], 1% Triton X-100, 0.5% Sodium deoxycholate, 0.1% SDS). The concentration of proteins was measured by bicinchoninic acid assay using Pierce™ BCA Protein Assay Kit (PIR-23227, Thermo Fisher Scientific), and a Click reaction was performed with Click-iT® Protein Reaction Buffer Kit (C10276, Thermo Fisher Scientific) according to manufacturer’s instructions.

## m^7^GTP pulldown assay

Cells were lysed (50 mM MOPS/KOH [pH 7.4], 100 mM NaCl, 50 mM NaF, 2 mM EDTA, 2 mM EGTA, 1% NP40, 1% Na-deoxycholate, protease inhibitors and phosStop), and incubated on ice for 15 minutes with occasional vortexing, then protein concentration was determined by bicinchoninic acid assay using Pierce™ BCA Protein Assay Kit (PIR-23227, Thermo Fisher Scientific). 500 μg of protein lysates were incubated with agitation for 1 hour at 4°C with 50 μl of immobilized γ-aminophenyl-m^7^GTP (C10-spacer)-Agarose beads (AC-155L, Jena Bioscience, Jena, Germany) pre-washed with washing buffer (50 mM MOPS/KOH [pH 7.4], 100 mM NaCl, 50 mM NaF, 0.5 mM EDTA, 0.5 mM EGTA, 7 mM beta-mercaptoethanol, 0.5 mM PMSF, 1 mM Na_3_VO_4_ and 0.1 mM GTP); the volume was completed to 1 ml with washing buffer. Beads were collected by centrifugation at 500 x g for 1 minute, washed 3 times with washing buffer, and the bound proteins were eluted from the beads by heating at 96°C for 10 minutes in 1X Laemmli sample buffer (50 mM Tris-Hcl [pH 6.8], 0.05% bromophenol blue, 8% Glycerol, 2% SDS, 2% β-mercaptoethanol). As a control, 75 μM of m^7^G(5')ppp(5')G RNA Cap Structure Analog (S1404S, New England Biolabs, Massachusetts) was pre-incubated with cell lysates for 1 hour at 4°C prior incubation with the m^7^GTP beads.

## Polysomes isolation and fractionation

To identify the transcripts under the translational regulation of 4EBPs, polysome profiling was done according to^3^. Cells were treated with 10 μg/ml of cycloheximide for 10 min, washed twice with PBS containing 100 μg/ml cycloheximide, then cells were scrapped and collected. Cells were pelleted by centrifugation (300 x *g*, 5 min, 4°C), lysed with 434 μl of lysis buffer (50 mM Tris-base [pH 8], 2.5 mM MgCl_2_, 1.5 mM KCl, 115 μg/ml cycloheximide, 2.3 mM DTT and 0.27 U/μl RNaseOUT [10777019, Thermo Fisher Scientific]), and vortexed. 25 μl of 100% Triton X-100 and 25 μl of 10% sodium deoxycholate were added to the cell lysates, which were vortexed and centrifuged (17,800 x *g*, 2 min, 4°C). 50 μl of the lysates were saved as the total fraction and the remaining were loaded on top of a three layers sucrose gradient (5%, 34% and 55% sucrose) that were prepared by dissolving sucrose in gradient buffer (4 mM HEPES [pH 7.6], 20 mM KCl, 1 mM MgCl_2_). The lysates loaded on top of the sucrose gradient were subjected to ultracentrifugation (229884 xg, 2.5 hours, 4°C). The polysome profile was read using a piston gradient collector (Biocomp) fitted with a UV detector (Tirax). Three polysomal fractions were collected and placed in Trizol (Sigma-Aldrich Company, location). RNA was extracted from frozen fractions using manufacturer's instructions.

## RNA sequencing and data analysis

Total RNAseq was performed as in^4^. The 3’RNAseq kit (Lexogen) was used for library preparation. Sequencing was performed using NEXTseq500 at G-INCPM (Weizmann Institute). All samples were tested for quality control using the FASTQC program, adaptors as well as poor quality nucleotide were trimmed using the Trim-Galore (v0.4.2) program. All samples were then mapped to the human genome (GRCh38) to calculate gene abundance using the RSEM(v1.2.25) program implying the STAR(v2.5.3a) mapper indicating the strand-specific option. The gene abundance count data was then used to identify differentially expressed genes using DESeq2 R package. In order to estimate differentially expressed genes in total RNA samples, we used a simple contrast between HEK293 sh4EBPs and shSCR samples. To estimate differences in *translational efficiency* we used a statistical model that takes into account the paired samples (polysome - total) as described in the DESeq2 manual. We then used the likelihood ratio test (LRT) to identify genes that show different pattern of expression between the fraction levels (polysome and total) while looking at the cell lines level (sh4EBPs and shSCR), i.e. the interaction between the fraction and the cell lines. Finally, we calculated the *translational efficiency* as the normalized count (RLOG) ratio between polysomal vs. total in each cell line (sh4EBPs and shSCR) for each significant gene identified. Enrichment analysis was performed using the clusterprofiler R package with annotation obtained from Ensembl. Clustering analysis was performed using hierarchical clustering with the ‘Euclidean’ matric and ‘ward.D2’ method.

# References

1. Dowling, R. R. J. O. *et al.* mTORC1-mediated cell proliferation, but not cell growth, controlled by the 4E-BPs. *Science (80-. ).* **328**, 1172–1177 (2010).

2. Tsukumo, Y., Sonenberg, N. & Alain, T. Transcriptional induction of 4E-BP3 prolongs translation repression. *Cell Cycle* **0**, 1–2 (2016).

3. Liang, S. *et al.* Polysome-profiling in small tissue samples. *Nucleic Acids Res.* **46**, 1–13 (2017).

4. Mazor, G. *et al.* The lncRNA TP73-AS1 is linked to aggressiveness in glioblastoma and promotes temozolomide resistance in glioblastoma cancer stem cells. *Cell Death Dis.* **10**, 246 (2019).
