# Supplementary figures and images for: 4EBP1/2 are active under standard cell culture conditions to regulate the translation of specific mRNAs
Source: Cell Death Dis. 2020 Nov 11;11(11):968. doi: 10.1038/s41419-020-03182-6 (PMC7659004; doi:10.1038/s41419-020-03182-6)

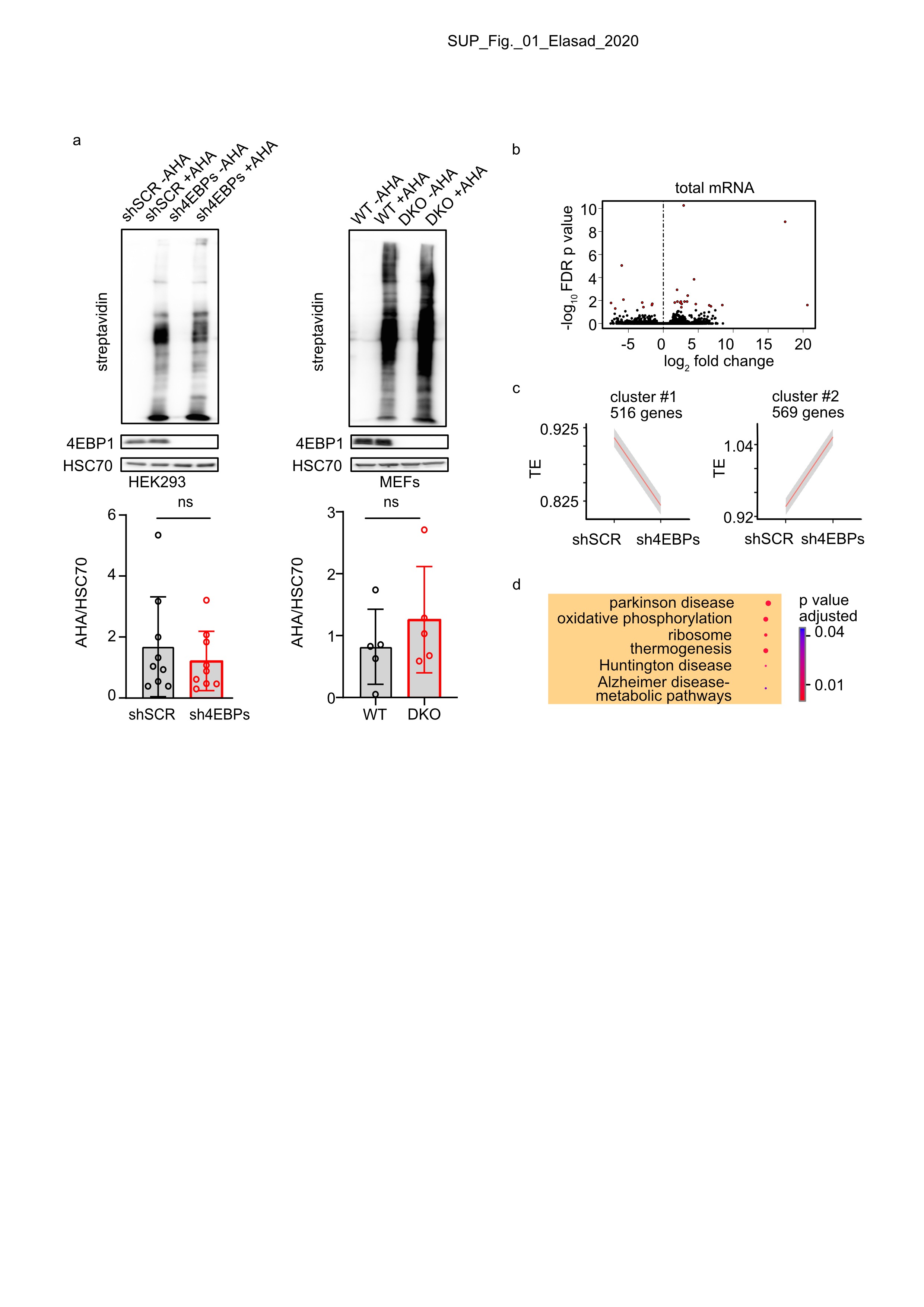

Supplement: Supplementary file 2 — Supplementary figure [file 41419_2020_3182_MOESM2_ESM.tif]
